# Fisetin Attenuates Myocardial Ischemia-Reperfusion Injury by Activating the Reperfusion Injury Salvage Kinase (RISK) Signaling Pathway

**DOI:** 10.3389/fphar.2021.566470

**Published:** 2021-03-08

**Authors:** Karthi Shanmugam, Sri Rahavi Boovarahan, Priyanka Prem, Bhavana Sivakumar, Gino A Kurian

**Affiliations:** ^1^ School of Chemical and Biotechnology, SASTRA Deemed University, Thanjavur, India; ^2^ Vascular Biology Lab, SASTRA Deemed University, Thanjavur, India

**Keywords:** ischemia-reperfusion injury (I/R), fisetin (PubChem CID, 5281614), wortmannin (PubChem CID, 312145), reperfusion injury salvage kinase (RISK) pathway, PI3K/Akt (PKB) axis, GSK3β = glycogen synthase kinase-3β, SB216763 GSK inhibitor, cardioprotection

## Abstract

Ischemia-reperfusion (I/R) injury is an unavoidable injury that occurs during revascularization procedures. In the previous study, we reported that fisetin is a natural flavonoid that attenuat**es** I/R injury by suppressing mitochondrial oxidative stress and mitochondrial dysfunction. Though fisetin is reported as a GSK3β inhibitor, it remains unclear whether it attenuates myocardial ischemia by activating the phosphoinositide 3-kinase/protein kinase B (PI3K/Akt) pathway, thereby inhibiting the downstream GSK3β, or by directly interacting with GSK3β while rendering its cardioprotection. In this study, the research team investigate**s** the possible mechanism of action of fisetin while rendering its cardioprotective effect against myocardial I/R injury in rats. For this investigation, the team utilized two myocardial I/R models: Ligation of the left anterior descending artery and Langendorff isolated heart perfusion system. The latter has no neurohormonal influences. The PI3K inhibitor (Wortmannin, 0.015 mg/kg), GSK3β inhibitor (SB216763, 0.7 mg/kg), and fisetin (20 mg/kg) were administered intraperitoneally before inducing myocardial I/R. The result of this study reveals that the administration of fisetin decreases the myocardial infarct size, apoptosis, lactate dehydrogenase, and creatine kinase in serum\perfusate of the rat hearts subjected to I/R. However, the inhibition of PI3K with Wortmannin significantly reduced the cardioprotective effect of fisetin both in the *ex vivo* and vivo models. The administration of GSK3β inhibitor after the administration of fisetin and Wortmannin, re-establishing the cardioprotection, indicates the major role of PI3K in fisetin action. Changes in myocardial oxidative stress (level) and mitochondrial functional preservation of interfibrillar and subsarcolemmal mitochondria support the above findings. Hence, the team here reports that fisetin conferred its cardioprotection against I/R injury by activating the PI3K/Akt/GSK3β signaling pathway in rat hearts.

## Introduction

Ischemic heart disease remains a major threat to humans. The most effective strategy to manage ischemic heart disease is the prompt restoration of blood supply through coronary interventions (Eltzschig and Eckle, 2011). However, the surgical or non-surgical interventions during the revascularization procedure in the heart often lead to another unavoidable injury, myocardial reperfusion injury, otherwise called ischemia-reperfusion (I/R) injury (Piper et al., 1998). Critically, attenuating or suppressing I/R injury by mechanical or pharmacological agents is a challenge, due to its multi-factorial pathology, and the search for new therapeutic strategies is in high demand (Hausenloy et al., 2017).

Flavonoids are multi-faceted molecules that demonstrate antioxidant, anti-inflammatory, anti-apoptotic, and anti-platelet aggregation properties, thus making them an attractive choice of therapeutic agents to ameliorate I/R injury (Akhlaghi and Bandy, 2009). The positive impact of flavonoids on cardiac I/R injury has been demonstrated both *in vitro* and *in vivo*. Specifically, the natural flavonoid fisetin has been reported to confer cardio-protection against myocardial I/R injury. Fisetin has been shown to reinforce mitochondrial physiology by suppressing oxidative stress level and increasing mitochondrial biogenesis (Shanmugam et al., 2018; Rodius et al., 2020). Since fisetin is well tolerated in human subjects even at a higher concentration with a lack of apparent toxic effects, this natural flavonoid has great potentiality to be developed as a candidate drug for the management of I/R-induced myocardial tissue injury. However, such a translation (transformation) should be mechanism-driven to minimize its chances of failure in clinical trials (Dolgos et al., 2016). The exact cardio-protective signaling and mechanisms of fisetin remain poorly investigated.

The previous study demonstrated that fisetin rendered cardioprotection primarily by suppressing oxidative stress and improving mitochondrial dysfunction. Mechanistically, the cardioprotective effects of fisetin were believed to be mediated through the inhibition of glycogen synthase kinase-3β (GSK3β), deciphered via in silico studies (Shanmugam et al., 2018). But the effective response of GSK3β on mitochondria in mediating cardioprotection depends specifically on the net effect of the mitochondrial specific broad spectrum of cardioprotective signaling pathways that converge to the GSK3β. It is noteworthy that GSK3β can exhibit a biphasic response to certain cell types, like a neuron. In addition, many studies have shown that inhibition of GSK3β related to mitochondrial signaling is associated with the activation of a broad spectrum of cardioprotective signaling pathways. It has been demonstrated that inhibition of GSK3β can be the consequence of activation of one or more signal transduction pathways, including PI3K, MEK, PKA, PKB/Akt, PEG, and PKC (Sadat, 2009). Interestingly, GSK3β acts as an integration point for several pathways and plays a central role in transferring protective signals downstream to targets that are involved in the pathology of oxidative stress and apoptosis (Juhaszova et al., 2009). Hence, this complex regulatory network creates difficulties in identifying the exact mode of action of fisetin while rendering its cardioprotection against I/R injury.

There are shreds of evidence that pharmacological manipulation and up-regulation of pro-survival kinase cascades refer to the Reperfusion Injury Salvage Kinase (RISK) pathway, as an adjunct to reperfusion, that may protect the myocardium from lethal reperfusion-induced cell death by preserving mitochondria (Hausenloy and Yellon, 2004). The RISK pathway includes phosphatidylinositol 3-kinase/Akt (PI3K/Akt), extracellular signal-regulated kinase (ERK1/2), and the downstream target GSK3β. It has been reported that various endogenous and exogenous molecules like insulin, insulin-like growth factor-1 (IGF-1), transforming growth factor-B1 (TGF-B1), cardiotrophin-1 (CT-1), urocortin, atorvastatin, and bradykinin protect the heart by activating PI3K-Akt and/or Erk1/2 kinase cascades, when given (administered) at the commencement of reperfusion, following a lethal ischemic insult (Hausenloy and Yellon, 2004).

In this direction and in a continuation of the previous work, in the present study, the research team evaluated the possible pharmacological manipulation of fisetin on the RISK pathway via PI3K/Akt signaling cascade to explore its mode of action. The research team inhibited PI3K, as an upstream target of the RISK signaling pathway, with Wortmannin and assessed the effect of fisetin on cardioprotection. Our result shows that the inhibition of the RISK signaling pathway significantly blunts the effect of fisetin on cardioprotection in an I/R-induced rat heart. Here, the team provides the first insights that fisetin activates RISK, as a pro-survival signaling pathway while rendering its cardioprotection against I/R injury.

## Materials and Methods

### Animals

Male Wistar rats, 8 weeks old and with an average weight of 220 g, were purchased from the central animal facility at SASTRA Deemed University. Rats were caged in a temperature-controlled room at (22 ± 2°C) with the relative humidity (60 ± 5%). All the experimental animal protocols followed in this research were reviewed and approved by the Institutional Animal Ethics Committee (IAEC), SASTRA University, Thanjavur, India (CPCSEA approval number: 552/SASTRA/IAEC/RPP). This study was conducted in accordance with the guidelines mentioned by the Committee for the Purpose of Control and Supervision of Experiments on Animals (CECSEA). All the experimental groups were exposed to a 12 h light/dark cycle with *ad libitum* water and feed supply.

### *Ex vivo* Perfusion Groups

Rats were randomly divided into seven groups and each experimental group contained six animals: 1) Normal; 2) Fisetin-Control (20 mg/kg, injected intraperitoneally) (Zhen et al., 2012) (procured from TOCRIS Bioscience, Bristol, United Kingdom); 3) Ischemia-Reperfusion (IR); 4) Fisetin-IR; 5) Wortmannin-IR (Wortmannin 0.015 mg/kg, injected intraperitoneally) (Wu et al., 2010); 6) Wortmannin-Fisetin-IR (0.015 mg/kg Wortmannin administered 30 min before the administration of 20 mg/kg fisetin intraperitoneally); and 7) Wortmannin-Fisetin-SB216763-IR (0.015 mg/kg Wortmannin administered, and after 30 mins, 20 mg/kg fisetin was administered followed by 0.7 mg/kg SB216763 (Zhang et al., 2020) 10 min before the excision of the heart). Langendorff isolated heart perfusion technique was used to induce I/R (Shanmugam et al., 2018). Rats were anaesthetized with sodium thiopentone (80 mg/kg, i.p.) 30 min after heparin treatment (500 IU/kg). The heart was rapidly excised and perfused with Krebs-Henseleit (KH) solution containing (in mM) NaCl (118.5), KCl (5.8), KH_2_PO_4_ (1.2), MgSO_4_ (1.2), CaCl_2_ (2.5), glucose (11), and NaHCO_3_ (25) in Langendorff perfusion apparatus maintained at 37°C with continuous carbogen (95% O_2_ + 5% CO_2_).

We followed the standard protocol used for inducing I/R, which consisted of 30 min of ischemia induced by stopping the buffer flow, followed by 60 min of reperfusion induced by resuming the flow. Till the end of the *ex vivo* experimental procedures, the hemodynamic changes, such as heart rate (HR), left ventricular end-diastolic pressure (LVEDP), and left ventricular developed pressure (LVDP), were recorded and calculated using Lab Chart Pro software (ADInstruments Inc., Sydney, Australia). At the end of the reperfusion, the perfusate was collected and used for further biochemical analysis. At the end of the experiment, the hearts were immediately frozen in liquid nitrogen and stored at −80°C for further analysis. For biochemical analysis associated with mitochondrial analysis, the tissues were homogenized in buffer containing 220 mM mannitol, 70 mM sucrose, 5 mM MOPS, 1 mM EDTA, and 0.2% BSA, at a pH of 7.4.

### mRNA Expression of PI3K Pathway Genes

The total RNA was extracted using the TRIzol reagent (Thermo Fisher Scientific, MA, United States) according to the manufacturer’s protocol. Using the Verso cDNA synthesis kit (Thermo Fisher Scientific) the reverse transcription was performed. Real-time PCR reactions were carried out using the DyNAmo Flash SYBR Green qPCR kit (Thermo Fisher Scientific) on a PCR system (ABI 7500, Applied Biosystems, Foster City, CA, United States). The amplification conditions were as follows: initial denaturation at 95°C for 2 min, followed by 35 cycles at 95°C for 30 s and 60°C for 30 s. The primers used in this study are listed below.

PI3K forward: 5′ GCA​TCA​GTG​GCT​CAA​GGA​CAA​G 3′

PI3K reverse: 5′CAA​GAT​AAA​GGT​TGC​CAC​GCA​GT3′

Akt forward: 5′AGG​AGG​AGG​AGG​AGA​TGG​A3′

Akt reverse: 5′GGT​CGT​GGG​TCT​GGA​AAG3′

β-actin forward: 5′TGA​CGA​TAT​CGC​TGC​GCT​C3′

β-actin reverse: 5′CAG​TTG​GTG​ACA​ATG​CCG​TG3′

### Western Blot Analysis

The myocardial left ventricle tissue samples were homogenized with an ice-cold RIPA lysis buffer. Protein was estimated using Lowry’s method and proteins of equal concentrations in SDS lysis buffer were denatured at 80°C for 15 min. The protein samples were then resolved in 5% stacking and 10% resolved SDS-PAGE gel and transferred on to 0.45 μm PVDF membranes. The membranes were blocked with 5% Bovine serum albumin in Tris-buffered saline with 0.1% Tween (TBST) for 1 h, washed thrice with TBST for 15 min, and then probed with primary antibodies (p-PI3K (Tyr 458/Tyr 199) (CST #4228), *p*-AKT (Ser 473) (CST #4060), Total Akt (CST #C67E7), Total PI3K (CST #4257), and Beta-actin (CST # 13E5)) at 4°C overnight. The membranes were incubated with anti-rabbit secondary antibody (CST #7074) in TBST at room temperature for 1 h after being washed thrice with TBST. The blot membranes were visualized and imaged on Chemi-Doc XRS (BioRad, United States of America) using chemiluminescent detection system (ECL, BioRad, United States) after washing three times. The relative band intensities were measured by image analysis software Quantity-One (BioRad, United States).

### Determination of Infarct Size

Immediately after the I/R procedure, hearts from each group were stained with 2,3,5-triphenyl tetrazolium chloride (TTC) to determine the area of the infarct regions, as described previously (Liu et al., 2009). The unstained TTC negative regions were quantified using ImageJ software (NIH, United States of America).

### Estimation of Injury Markers

The cardiac injury was assessed by analyzing the enzymatic activities of the injury marker enzymes lactate dehydrogenase (LDH) and creatine kinase (CK). Enzymatic activities of these enzymes were analyzed in perfusate, plasma, and tissue samples of the different experimental groups as previously described by our group (Ravindran et al., 2017b). The LDH activity was measured by estimating the conversion of lactate to pyruvate by monitoring the absorbance of NADH at 340 nm. The CK activity was measured by estimating the concentration of the inorganic phosphate, which is liberated during the conversion of ATP, quantified using reagent, and estimated at 660 nm. Apoptosis was determined by measuring the Caspase-3 enzyme activity, using a method already reported by Denton et al. (2009). Protein content was determined by the Bradford method using Bio-Rad reagent with bovine serum albumin (BSA) as a standard.

### Isolation of Mitochondrial Subpopulations

The mitochondrial subpopulations from the rat hearts were isolated using the differential centrifugation method as described previously (Palmer et al., 1985). The crude mitochondrial pellet was subjected to 12,000 × g for 10 min at 4°C to isolate SSM. The homogenate pellet from the previous step (after removing the supernatant for crude mitochondrial fraction) was incubated in trypsin (0.5 mg/g) to disrupt the fibers and release the IFM. The pellet was suspended in the isolation buffer and subjected to centrifugation at 600 g for 10 min at 4°C. The supernatant was collected and centrifuged at 12,000 × g for 10 min at 4°C to pellet the IFM. The pelleted mitochondrial subpopulations were suspended in storage buffer containing (in mM) 25 sucrose, 75 sorbitol, 10 Tris-HCl, 100 KCl, 10 K_2_HPO_4_, 5 MgCl_2,_ and 0.05 EDTA, adjusted to pH 7.4 and stored at 4°C until further analysis.

### Mitochondrial Electron Transport Chain Complex Activity

Mitochondrial oxidative phosphorylation system (OXPHOS) was assessed by measuring ETC enzyme activities in the IFM and SSM subpopulations independently. As described earlier by Frazier and Thorburn (2012), the spectrophotometry-based ETC assay analysis was carried out by using the assay-specific donor-acceptor assay. Rotenone-sensitive NADH-oxidoreductase (NQR), succinate decylubiquinone DCPIP reductase (SQR), Ubiquinol cytochrome c reductase (QCR), and cytochrome c oxidase (COX) activities were measured.

### Antioxidant Enzymes

The antioxidant status of IFM and SSM was evaluated by measuring GSH and GSSH level using Ellman’s reagent, as reported by Sedlak and Lindsay (1968), superoxide dismutase (SOD) as reported by Nandi and Chatterjee (1988), and the catalase activity as reported by Goldblith and Proctor (1950).

### LAD Ligation Model

Myocardial infarction was induced by ligation of the left anterior descending (LAD) coronary artery, as reported by us previously (Ravindran et al., 2017a). In brief, rats were anesthetized using an inhalation anesthetic: halothane (1.5% with O_2_) (Vogler, 2006). Rats were supported on a rodent ventilator (70 strokes/min at a tidal volume of 10 ml/kg) and placed on a thermal heating pad to maintain 37°C. After 10 min of the stabilization period, an incision was made between the 3^rd^ and 4^th^ intercostal ribs to locate the heart. To locate the left anterior descending artery, the pericardium was detached. A 7–0 suture was passed below the LAD, and a slipknot was made to occlude the blood flow. Ischemia was created for 30 min, which was confirmed by the appearance of regional epicardial cyanosis, and the knot was released for reperfusion of the myocardium for 2 h. Successful reperfusion was visualized from the arterial blood flow and the over-the-surface appearance of the previous segment (Xu et al., 2014).

### Experimental Group

The rats were divided randomly into six groups and each experimental group contained six animals 1) Normal; 2) Fisetin-Control (20 mg/kg, injected intraperitoneally) (Zhen et al., 2012) (TOCRIS Bioscience, Bristol, United Kingdom); 3) Ischemia-Reperfusion (IR); 4) Fisetin-IR; 5) Wortmannin-IR (Wortmannin 0.015 mg/kg, injected intraperitoneally) (Wu et al., 2010); and 6) Wortmannin-Fisetin-IR (0.015 mg/kg Wortmannin administered 30 min before the administration of 20 mg/kg fisetin intraperitoneally). During the end of reperfusion, blood was collected in K_2_EDTA tubes, and the heart was isolated and immediately flash-frozen in liquid nitrogen for storage at −80°C until further analysis. For the biochemical analysis, the tissues were homogenized in 0.1N Tris HCl buffer, pH 7.4, and, for the mitochondrial analysis, tissues were homogenized in isolation buffer. Since isolated rat heart model is devoid of neuro systemic influence, heart tissues from an LAD model were used to assess cardiac injury (to account for the inflammatory component), mitochondrial enzyme activity, and corresponding oxidative stress. Blood analysis was performed to confirm the cardiac injury. Cardiac Troponin I ELISA Kit (ARG82123) kit used to analyze the serum troponin T level.

### Histopathological Examination of Ventricle Sections

For histopathological evaluation, heart tissues were fixed in 10% neutral buffered formalin and embedded in paraffin to make tissue blocks. The blocks were then cut into 5 μm sections and stained with hematoxylin and eosin (H&E). At least three hearts from each group were examined under a light microscope (NIKON, JAPAN) for any pathological changes.

### Statistical Analysis

Statistical analysis was carried out by GraphPad Prism (version 8.00) for Macintosh, GraphPad Software, La Jolla California USA (www.graphpad.com). Experimental results were expressed as mean ± SD. Comparison between groups was made using analysis of variance (ANOVA) with post hoc test. ANOVA was performed for multiple comparisons between groups using a two-way comparison of means by Dunnett tests, and a *p*-value of <0.05 was considered to be statistically significant.

## Results

The underlying mechanism of fisetin-mediated cardioprotection was evaluated using two experimental approaches, which include regional I/R using LAD and global I/R using Langendorff isolated rat heart model, where the latter experimental model will not account for any neurohormonal influences in the cardiac performance.

### Fisetin Treatment Mediates the Activation of the RISK Signaling Pathway by Upregulating PI3K Gene Expression in the LAD Model

**FIGURE 1:**
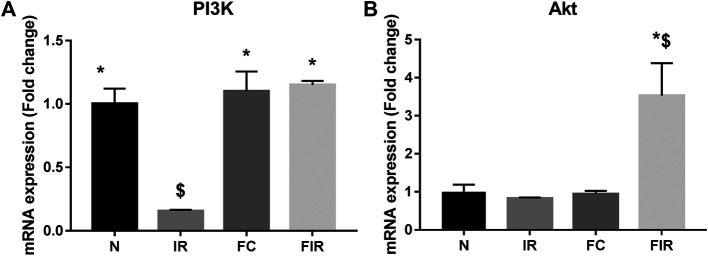
Effect of fisetin on the PI3k/Akt signaling pathway determined by qRTPCR technique. **(A)** mRNA expression changes of PI3k **(B)** mRNA expression changes of Akt (N, Normal; IR, Ischemia-reperfusion; FC, Fisetin-Control; FIR, Fisetin-IR). Data were represented as mean ± SD. *p < 0.05 vs. IR, $ *p* < 0.05 vs. N (*n* = 6 per group).

**FIGURE 2:**
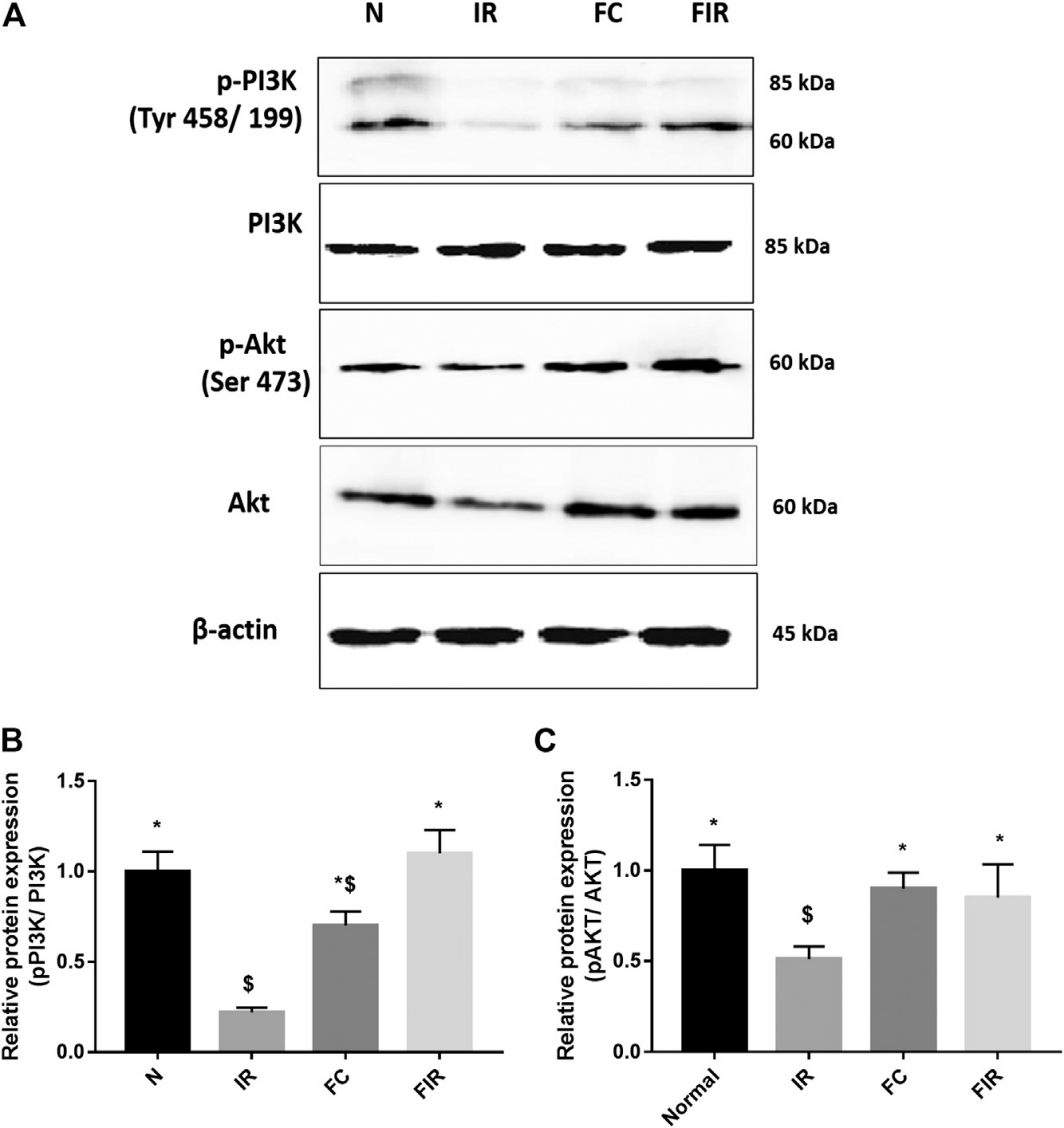
Effect of fisetin on the PI3k/Akt signaling pathway determined by blot. **(A)** Representative blot images of p-PI3k, Total PI3K, *p*-AKT (Ser 473), Total AKT, and *ß* actin proteins, **(B)** Relative protein expression of p-PI3k/PI3K, **(C)** Relative protein expression of *p*-AKT/AKT (N, Normal; IR, Ischemia-reperfusion; FC, Fisetin-Control; FIR, Fisetin-IR) Data were represented as mean ± SD. *p < 0.05 vs. IR, $ *p* < 0.05 vs. N.

To investigate the mechanism of action behind fisetin-mediated cardioprotection, we evaluated the modulation of PI3K and AKT signaling molecules (involved in RISK cardioprotective signaling pathway) during fisetin-mediated cardioprotection. We analyzed the mRNA gene expression of PI3K and Akt genes in the myocardium and found that the PI3K gene expression was significantly diminished in I/R rat hearts and treatment of the heart with fisetin upregulated the mRNA expression level of both PI3K and AKT (Figure 1). Unlike in the normal myocardium, I/R induced a decreased protein expression of p-PI3K and p-AKT (Ser473), which was improved by fisetin treatment (Figure 2).

### Effect of Fisetin on I/R Induced Cardiac Injury in the Presence of PI3K Inhibitor

**FIGURE 3:**
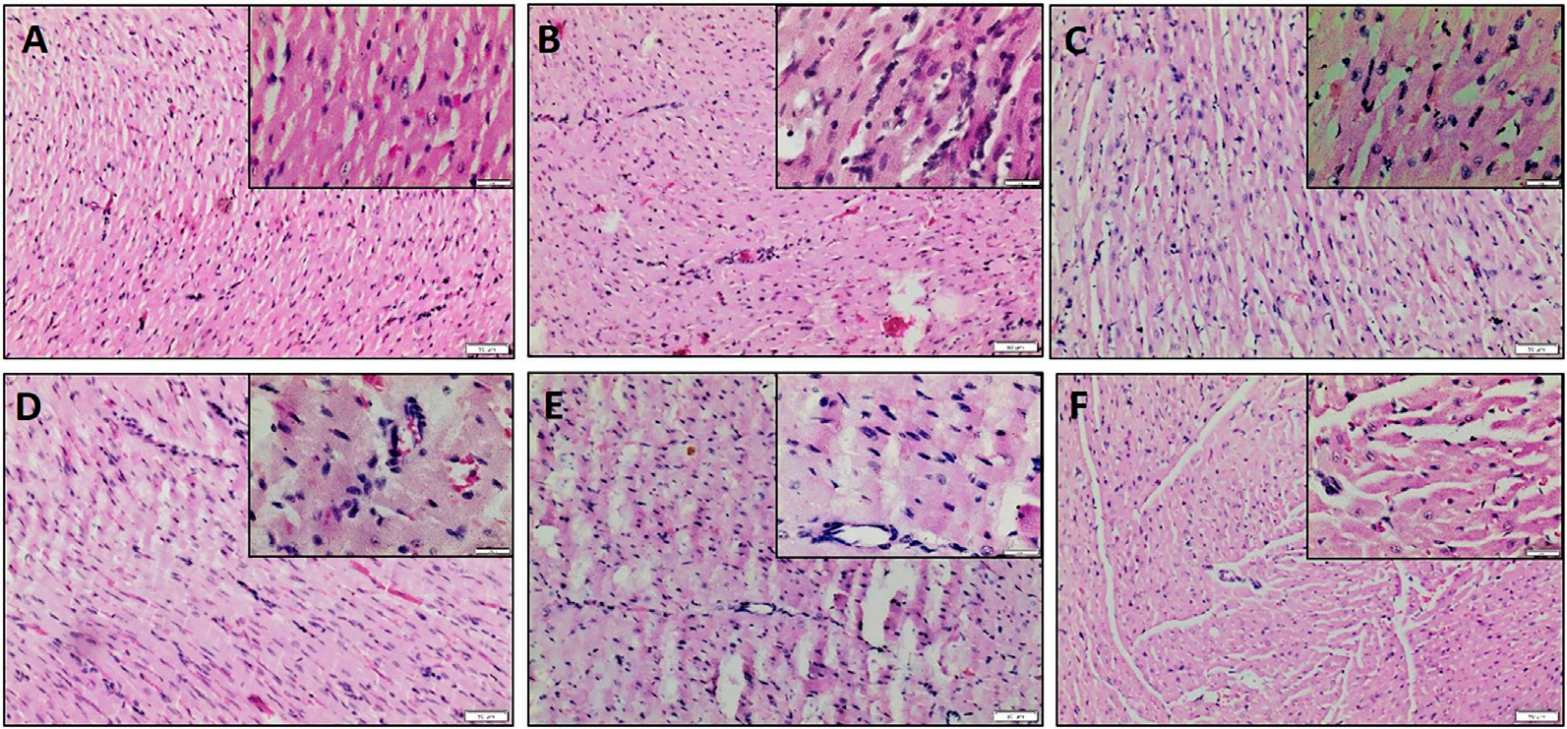
H&E-stained myocardial sections. **(A)** Normal heart showing normal histological architecture of cardiomyocyte which appear longitudinally and obliquely cut, **(B)** IR heart shows unclear arrangement, oedema, and neutrophil infiltration, **(C)** Fisetin Ctrl exhibiting normal myocardial histology, **(D)** Fisetin IR showing reduced neutrophil infiltration and oedema, **(E)** Wortmannin IR and **(F)** Fisetin_Wortmannin IR showing loss of architecture, wavy fibers, increased interstitial oedema, and neutrophil infiltration. Scale bars: 50 μm (lower magnification, 100X) and 20 μm (higher magnification, 400X, Insight).

**FIGURE 4:**
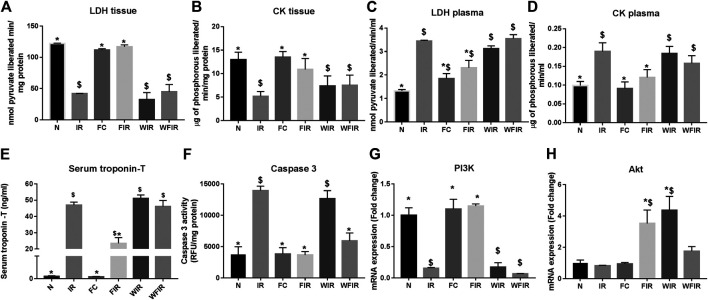
Effect of fisetin on the cardiac injury markers in myocardium subjected to I/R using LAD: the cardiac injury markers, namely lactate dehydrogenase, creatine kinase, cardiac troponin T, and caspase-3 activities, were evaluated from the plasma and myocardium to assess the extent of injury in different study groups. **(A)** LDH in tissue, **(B)** LDH leakage in plasma, **(C)** CK in tissue, **(D)** CK leakage in plasma, **(E)** Serum troponin – T, **(F)** Caspase-3 activity as a measurement of apoptosis, **(G)** mRNA expression changes of PI3k, **(H)** mRNA expression changes of Akt (N, Normal; IR, Ischemia-reperfusion; FC, Fisetin-control; FIR, Fisetin-IR, WIR, Wortmannin-IR; WFIR, Wortmannin-Fisetin-IR) Data were represented as mean ± SD. *p < 0.05 vs IR, $*p* < 0.05 vs. N (*n* = 6 per group).

The influential role of the PI3K pathway on fisetin-mediated cardioprotection in I/R-induced cardiac injury was assessed by regional and global I/R models. Most of the existing novel cardio-protective strategies are linked to neural pathways or endogenous cytoprotective blood-borne substances that can mask the direct pharmacological action of the drug on the heart. In order to overcome this problem, we used the isolated rat heart model. In both models, fisetin-mediated cardioprotection was lost in the presence of Wortmannin. The results from the LAD model showed a deteriorated arrangement of myofibrils in the myocardium, the presence of interstitial oedema, and increased hemorrhage and neutrophil infiltration in the Fisetin_Wortmannin IR group as shown in Figure 3F, as well as elevated levels of LDH, CK, and troponin T in the plasma and myocardial caspase 3 level, when compared with the normal group (Figure 4).

**FIGURE 5:**
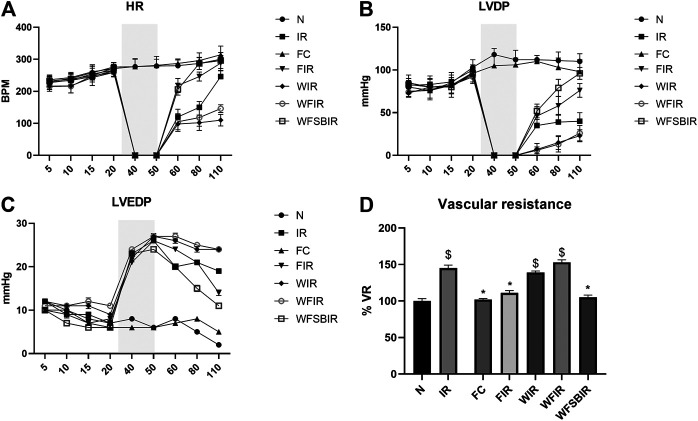
Fisetin fails to recover the hemodynamic function of Langendorf-perfused rat hearts submitted to I/R in the presence of wortmannin. **(A)** HR-heart rate changes across the time points, **(B)** LVDP-left ventricular developed pressure changes across the time points, **(C)** LVEDP-Left ventricular end diastolic pressure changes across the time points, **(D)** Bar graph represents the changes in the mean of vascular resistances (VR, mmHg*min/ml). Values were taken 60 min after reperfusion and were normalized to controls’ values. Grey colored area represents the ischemic time. Data were represented as mean ± SD. *p < 0.05 vs. IR, $*p* < 0.05 vs. N (*n* = 6 per group).

**FIGURE 6:**
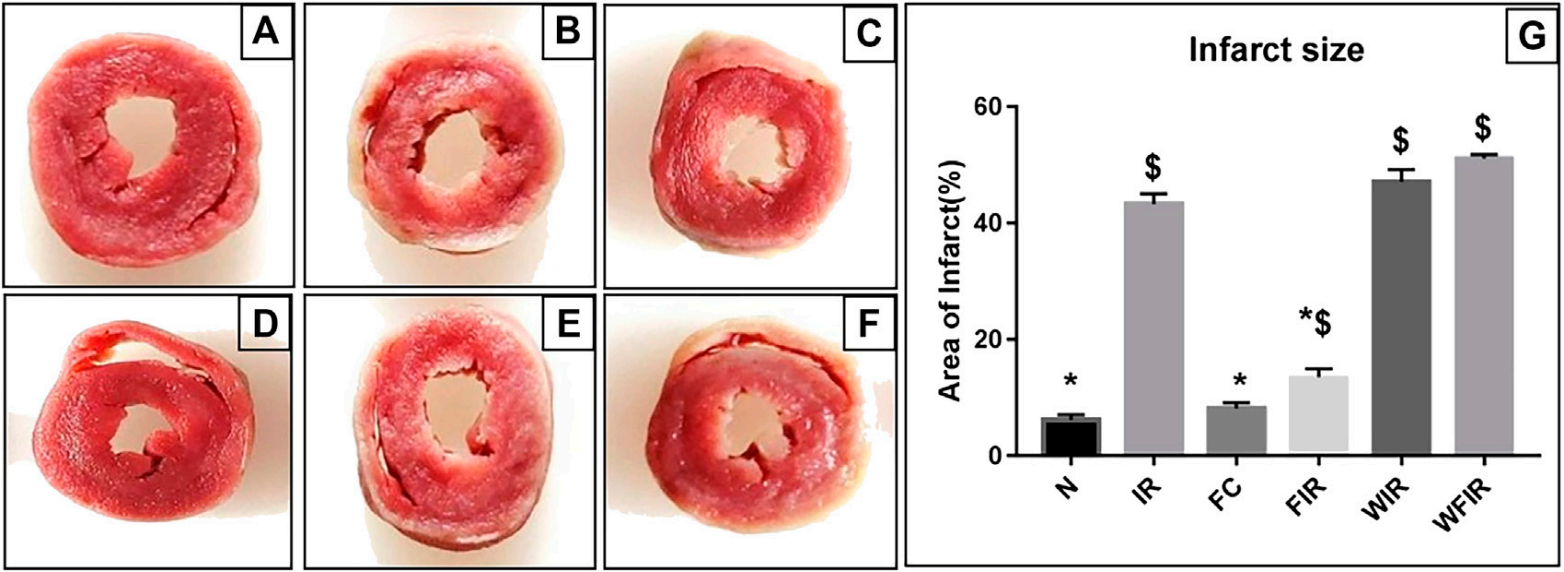
Effects of fisetin on ischemia/reperfusion injury-induced myocardial infarction in rats. 2,3,5-triphenyltetrazolium chloride (TTC) staining was used to determine the ischemic size and infarction size. **A**. The normal area was stained red, and the area of infract size (IS) was stained pale white. **(A)** Normal; **(B)** IR; **(C)** Fisetin-ctrl; **(D)** Fisetin-IR; **(E)** Wortmannin-IR; **(F)** Wortmannin-fisetin-IR; **(G)** Area of infarct. (N, Normal; IR, Ischemia-reperfusion; FC, Fisetin-Control; FIR, Fisetin-IR, WIR, Wortmannin-IR; WFIR, Wortmannin-Fisetin-IR). Results are expressed as mean ± SD (*n* = 3 per group). *p < 0.05 vs. IR, $ *p* < 0.05 vs. N (*n* = 6 per group).

**FIGURE 7:**
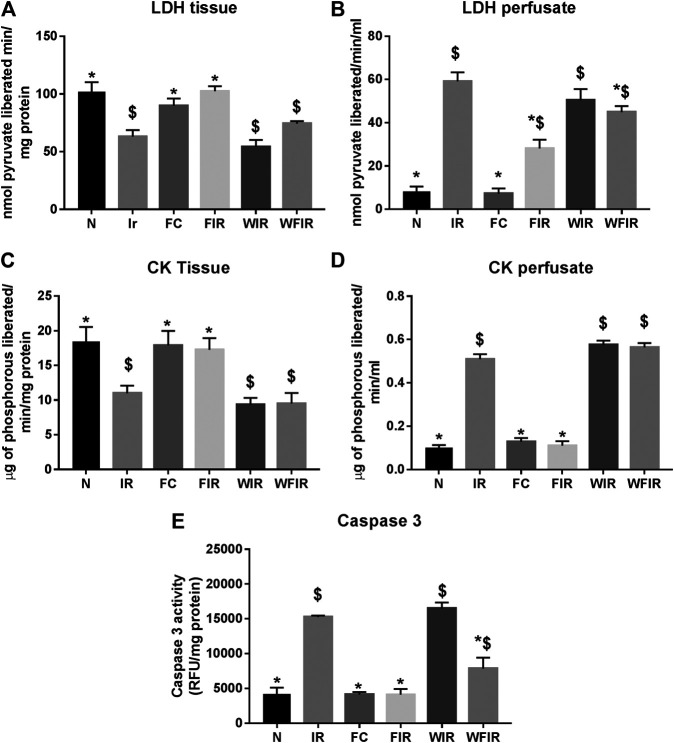
Effect of fisetin on the cardiac injury markers in myocardium subjected to I/R in isolated rat heart: the activities of the cardiac injury markers, namely lactate dehydrogenase, creatine kinase, and caspase-3, were evaluated from the coronary perfusate and myocardium to assess the extent of injury in different study groups. **(A)** LDH in tissue **(B)** LDH leakage in perfusate **(C)** CK in tissue **(D)** CK leakage in perfusate **(E)** Caspase-3 activity as a measurement of apoptosis. (N, Normal; IR, Ischemia-reperfusion; FC, Fisetin-control; FIR, Fisetin-IR, WIR, Wortmannin-IR; WFIR, Wortmannin-Fisetin-IR). Data were represented as mean ± SD. *p < 0.05 vs. IR, $*p* < 0.05 vs. N (*n* = 6 per group).

This result was supported by the elevated cardiac injury in isolated rat heart model. The effects of fisetin (20 mg/kg) on hemodynamic parameters (LVDP, HR, and LVEDP) in different experimental groups were depicted in Figure 5 and Supplementary Table S1. The hearts subjected to 30 min of ischemia, followed by 60 min of reperfusion, showed a significant decrease in LVDP(63%), and an increase in the values of LVEDP(89%) and VR (31%) from the normal sham control heart. Compared with I/R hearts, fisetin pretreatment improved the cardiac hemodynamic index, evident by the increase in left ventricular developed pressure (LVDP)(47%) and decrease in LVEDP(26%) and VR(23%), and the values were close to the normal level. But fisetin had the least effect on the cardiac physiological responses, in the presence of PI3K inhibitor Wortmannin, assessed by the decreased recovery in LVDP and a further increase in LVEDP and VR by 35%, 20%, and 5% respectively, compared with IR group. TTC staining (Figure 6) and cardiac injury markers in the perfusate were used to evaluate the efficacy of fisetin to ameliorate I/R injury in the presence of Wortmannin (Figure 7)) (Infarct size: IR = 42%, Wortmannin-Fisetin-IR = 52%; LDH: IR = 59.1 ± 4.12, Wortmannin-Fisetin-IR = 44.9 ± 6.2; CK: IR = 0.50 ± 0.023, Wortmannin-Fisetin-IR = 0.56 ± 0.02) and in the absence of Wortmannin (Infarct size: IR = 42%, fisetin-IR = 15%; LDH: IR = 59.1 ± 4.12, Fisetin-IR = 28.09 ± 2.08; CK: IR = 0.50 ± 0.023, Fisetin-IR = 0.11 ± 0.01). The results showed that the fisetin-IR group had recovered from I/R almost to the near-normal group level in the absence of wortmannin, but showed a significant difference from the normal group in the presence of wortmannin, similar to the IR group.

At mRNA level, treatment with fisetin reversed the IR-induced decreased mRNA expression level of PI3K in the absence of Wortmannin (in Fisetin-IR) but failed to exhibit the same in the presence of wortmannin (Wortmannin-Fisetin-IR group) (Figure 4G). Contrarily, Akt gene expression was found to be significantly upregulated in both Fisetin-IR and Wortmannin-Fisetin-IR rat hearts (Figure 4H).

### Impaired Cardioprotection by Fisetin in the Presence of Wortmannin was Reversed by a GSK3β Inhibitor

Since the inhibition of cardioprotective signaling pathway PI3K with Wortmannin resulted in the failure of fisetin-mediated cardioprotection, we next examined whether this failure could be reversed by triggering the downstream target of this pathway. In this line of thought, we inhibited the downstream protein GSK3β using SB216763, a GSK3β inhibitor. We administered 0.7 mg/kg of SB216763 intraperitoneally after administering 20 mg/kg of fisetin. The improved hemodynamics confirmed the reversal of Wortmannin-mediated resistance of fisetin’s cardioprotective effect, as shown in Figure 5.

### Effect of Fisetin on the Mitochondrial Functional Activity in the Presence of Wortmannin

**FIGURE 8:**
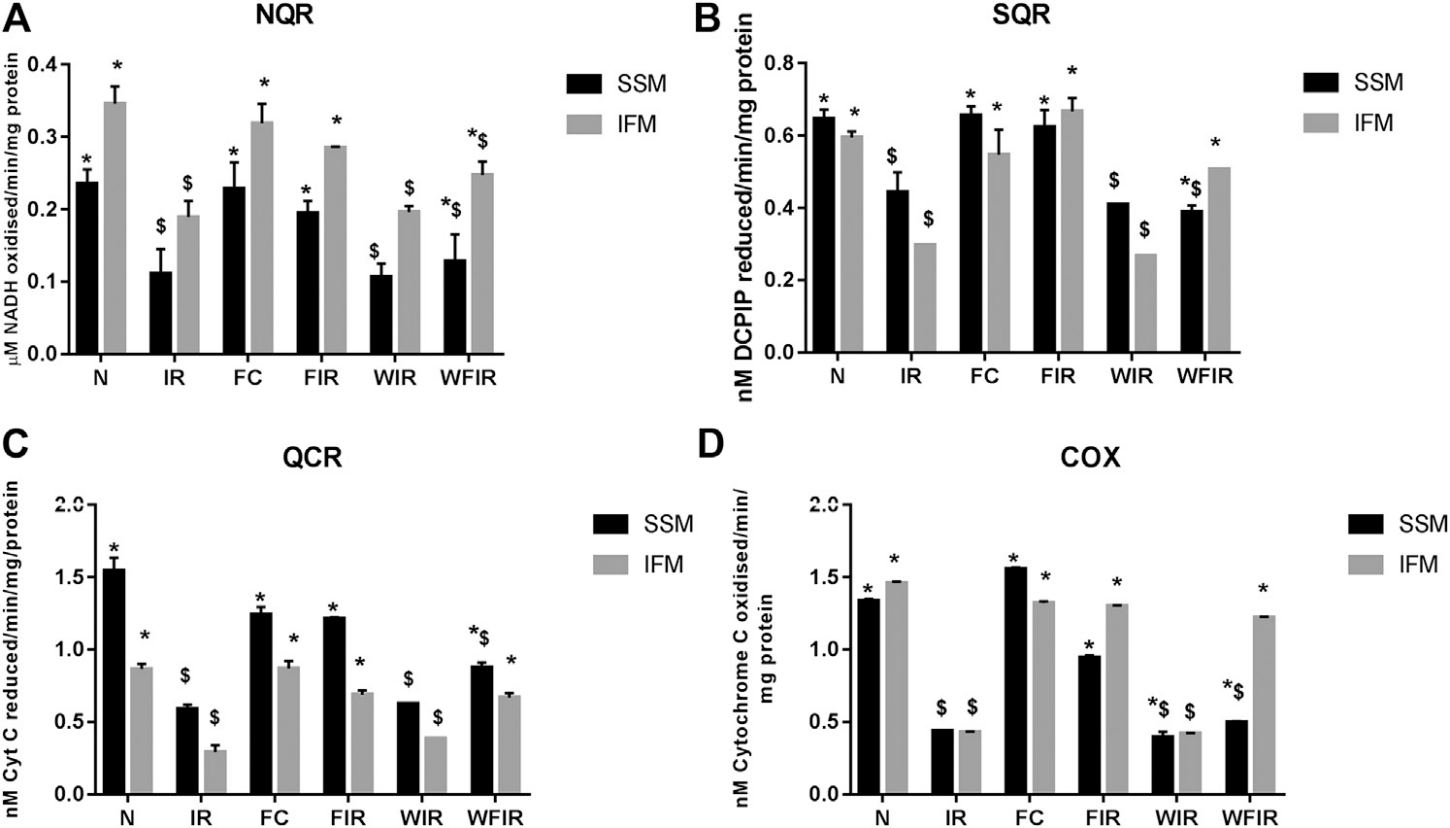
Effect of fisetin on mitochondrial enzyme activities in isolated rat heart model. **(A)** NQR (Rotenone-sensitive NADH-oxidoreductase), **(B)** SQR (succinate decylubiquinone DCPIP reductase), **(C)** QCR (Ubiquinol cytochrome c reductase) and **(D)** COX (cytochrome c oxidase) activities in the mitochondrial samples isolated from heart tissues. (N, Normal; IR, Ischemia-reperfusion; FC, Fisetin-Control; FIR, Fisetin-IR, WIR, Wortmannin-IR; WFIR, Wortmannin-Fisetin-IR). Data were represented as mean ± SD. *p < 0.05 vs respective IR, $*p* < 0.05 vs respective N. (*n* = 6 per group).

Evidence from the previous studies suggests that PI3K can affect mitochondrial function by augmenting the expression of peroxisome proliferator-activated receptor γ coactivators-1β, as an energetic regulator of cardiac metabolism (Gao et al., 2011). Similarly, in an another study, a direct effect of PI3K on mitochondrial K_ATP_ channel via GSK3β (Gross et al., 2007) was also reported. Thus, the inhibition of PI3K by Wortmannin is believed to modulate the mitochondrial functional activity. Accordingly, Figure 8 shows the impact of Wortmannin on the functional activity of mitochondria from the heart conditioned with fisetin. The result demonstrated that the reduced mitochondrial preservation by fisetin in I/R challenged rat heart was reduced in the presence of Wortmannin.

**FIGURE 9:**
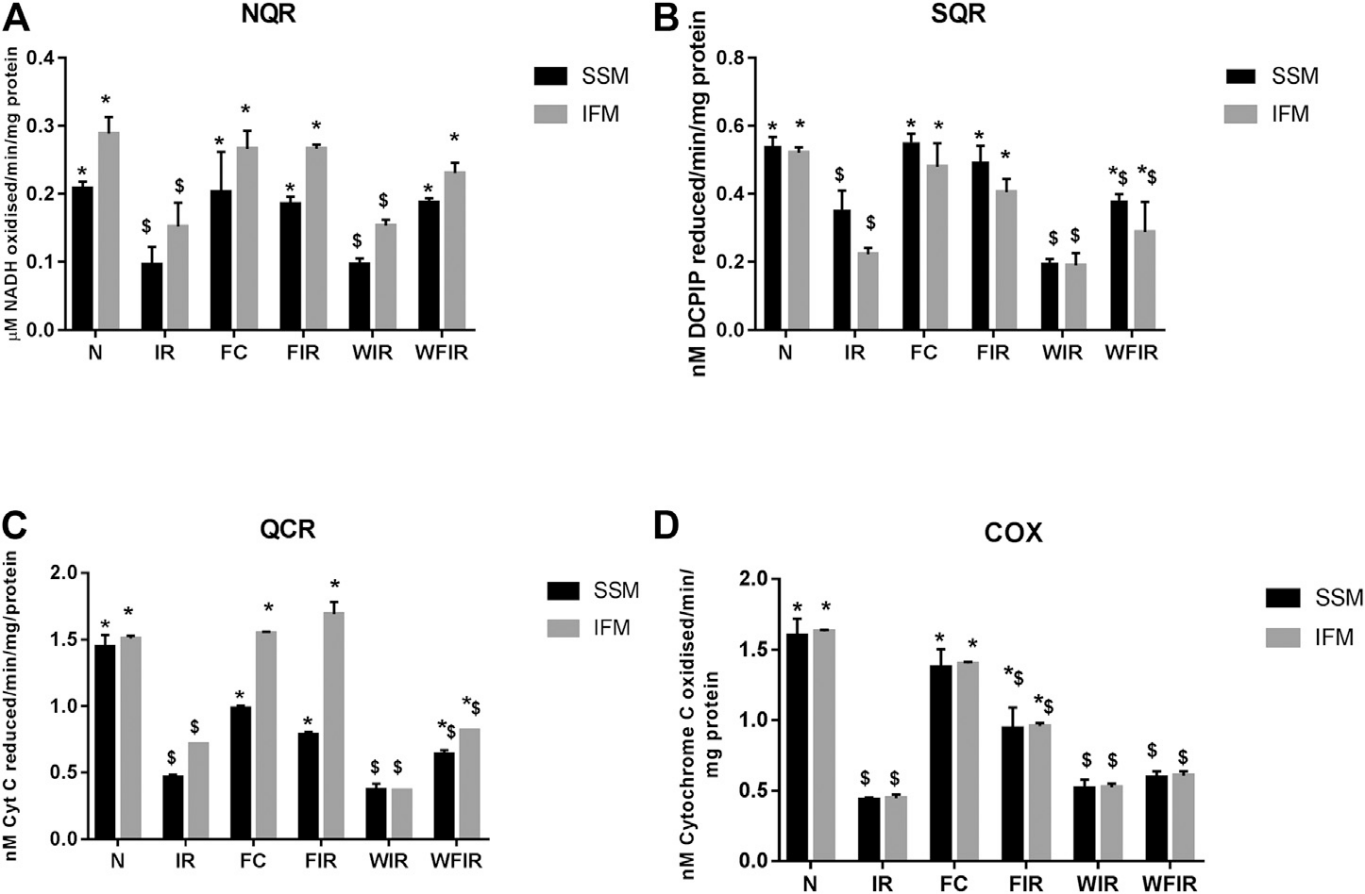
Effect of fisetin on mitochondrial enzyme activities. **(A)** NQR (Rotenone-sensitive NADH-oxidoreductase), **(B)** SQR (succinate decylubiquinone DCPIP reductase), **(C)** QCR (Ubiquinol cytochrome c reductase), and **(D)** COX (cytochrome c oxidase) activities in the mitochondrial samples isolated from LAD heart tissues. (N, Normal; IR, Ischemia-reperfusion; FC, Fisetin-Control; FIR, Fisetin-IR, WIR, Wortmannin-IR; WFIR, Wortmannin-Fisetin-IR). Data were represented as mean ± SD. *p < 0.05 vs. respective IR, $ *p* < 0.05 vs. respective N (*n* = 6 per group).

The treatment with fisetin had significantly improved the mitochondrial functioning by increasing ETC enzyme activities in isolated rat heart and brought the activity near to the normal level, as shown in Figure 8 [Wortmannin-Fisetin-IR SSM: 42% (NQR), 29% (SQR), 52% (QCR), 59% (COX); Wortmannin-Fisetin-IR IFM: 35% (NQR), 56% (SQR), 57% (QCR), 67% (COX). Similar effects of fisetin were observed in the mitochondria obtained from the LAD model as well (Figure 9). In order to study the role of the PI3K pathway on this fisetin-mediated mito-protection, the complex ETC enzyme activities were measured in the presence of Wortmannin prior to fisetin treatment in IR challenged rat. Accordingly, the data suggest that fisetin-mediated mito-protection was abrogated (the complex activities reduced to 8% (NQR), 13% (SQR), 6% (QCR), and 12% (COX) respectively in SSM and 25% (NQR), 42% (SQR), 23% (QCR), and 65% (COX) respectively in IFM]. A similar result was obtained with ETC enzymes of mitochondria from different experimental group of animals that underwent LAD protocol.

### Antioxidant Effect of Fisetin in the Presence of Wortmannin

**FIGURE 10:**
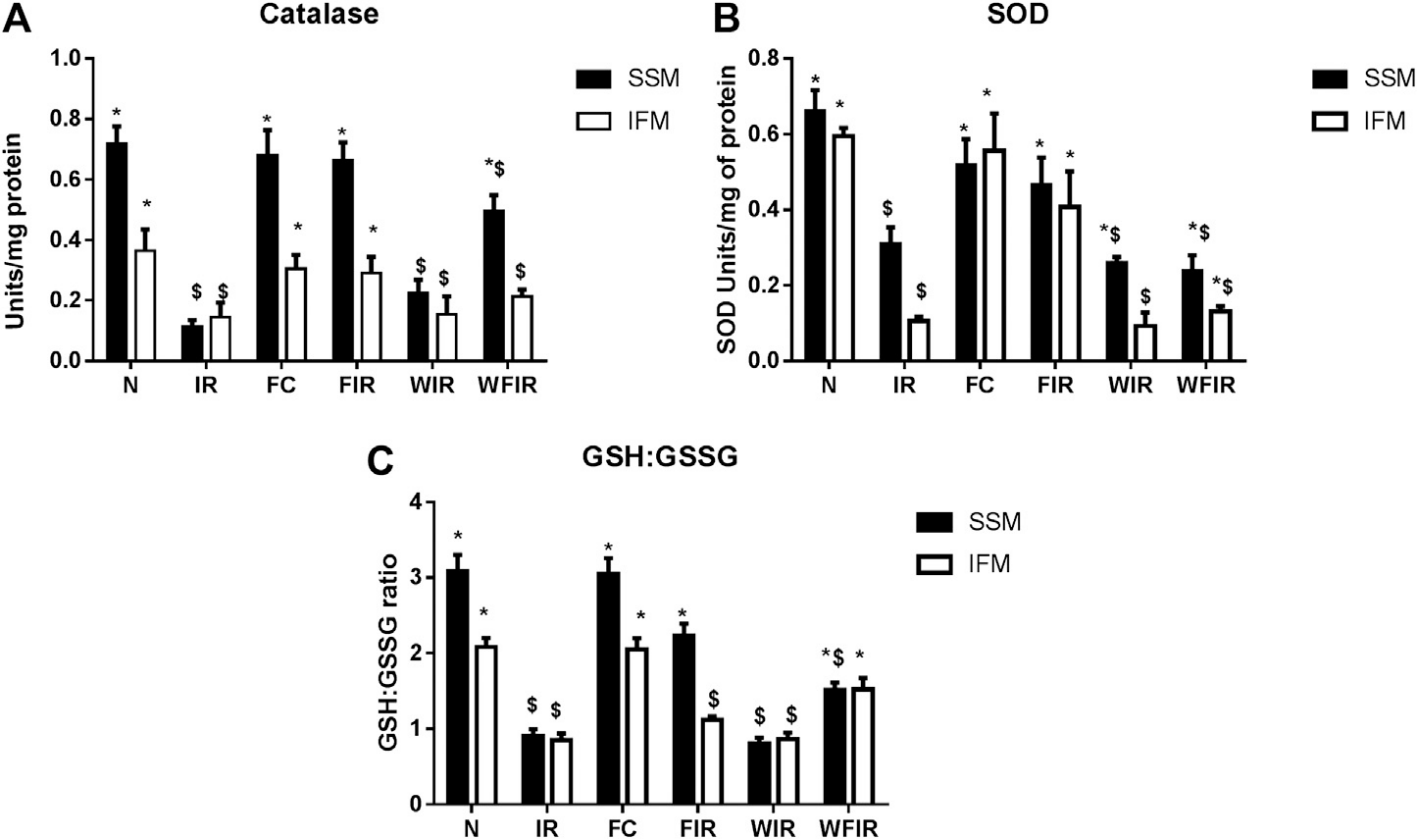
Oxidative stress markers. Effect of fisetin on antioxidant defense system isolated from LAD heart tissues. **(A)** Catalase activity, **(B)** SOD (Superoxide dismutase) activity, **(C)** GSH: GSSG activity in the respective groups. (N, Normal; IR, Ischemia-reperfusion; FC, Fisetin-Control; FIR, Fisetin-IR, WIR, Wortmannin-IR; WFIR, Wortmannin-Fisetin-IR). Data were represented as mean ± SD. *p < 0.05 vs. respective IR, $ *p* < 0.05 vs respective N (*n* = 6 per group).

**FIGURE 11:**
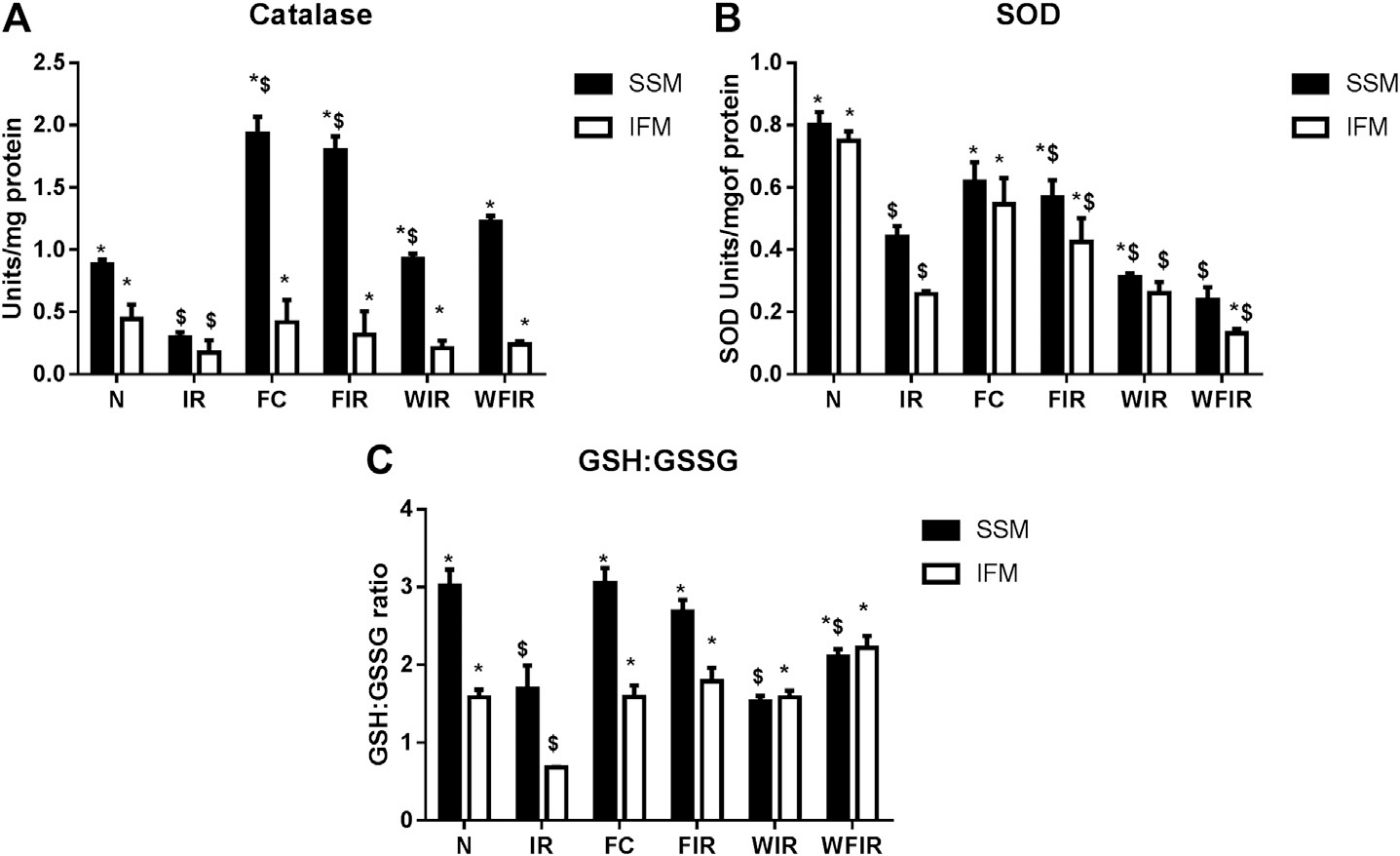
Oxidative stress markers. Effect of fisetin on antioxidant defense system isolated from heart tissues. **(A)** Catalase activity, **(B)** SOD (Superoxide dismutase) activity, **(C)** GSH: GSSG activity in the respective groups. (N, Normal; IR, Ischemia-reperfusion; FC, Fisetin-Control; FIR, Fisetin-IR, WIR, Wortmannin-IR; WFIR, Wortmannin-Fisetin-IR). Data were represented as mean ± SD. *p < 0.05 vs. respective IR, $*p* < 0.05 vs respective N (*n* = 6 per group).

Previous studies have established a direct relationship between PI3K signaling pathway modulation and increased cellular ROS level via altered mitochondrial bioenergetics and increased activity of NADPH oxidase (Koundouros and Poulogiannis, 2018). Once we found that Wortmannin declined the mitochondrial ETC enzyme activities in both subpopulations, we further evaluated the oxidative stress experienced by the organelle. As shown in Figure 10 and Figure 11, in both LAD and isolated rat heart model, I/R increased the oxidative stress significantly by decreasing the GSH/GSSG ratio, catalase, and SOD activities in both mitochondrial subpopulations, when compared to the normal group. The rat hearts treated with fisetin had significantly reduced the oxidative stress imposed by I/R even in the presence and absence of Wortmannin, emphasizing the possible free radical scavenging potential of fisetin, which is yet to be reported.

## Discussion

I/R injury is an unavoidable injury that occurs during revascularization procedures like coronary artery bypass graft (CABG) or percutaneous transluminal coronary angioplasty (PTCA). As I/R injury is associated with severe clinical manifestations of post-surgical patients, it is a major concern of surgeons in the management of ischemic heart patients (Piper et al., 1998). Unfortunately, to date, there are no clinically effective drugs to combat I/R pathology. In our previous study, we reported the potential of fisetin as a therapeutic agent to fight against I/R injury primarily due to its inherent ability to reduce oxidative stress and inflammatory responses (Shanmugam et al., 2018) without exploring its competency to modulate cardioprotective signaling pathways. The clear understanding of the underlying mechanism of fisetin at the molecular level is a prerequisite for its translation to clinical trials. Many natural polyphenols were reported to attenuate I/R injury in preclinical studies, but very few molecules are considered for clinical trials due to the lack of substantial evidence for its efficacy and toxicity at the molecular level.

Pharmacological manipulations of the pro-survival kinase pathway fetch many promising results toward the management of I/R injury in preclinical studies, where a few screened molecules are in clinical trials as well. In this study, we evaluated the cardioprotective effect of fisetin against I/R injury by probing the PI3K/AKT cell survival pathway. The outcome of the current study data demonstrated that the intraperitoneal administration of fisetin attenuated I/R injury via augmentation of the PI3K signaling pathway, which in turn stabilized the altered hemodynamic parameters, reduced infarct size, and preserved the cardiac mitochondria including IFM and SSM.

Studies from the literature pointed out multiple factors involved in the pathology of I/R injury and that are under the control of multiple complex signaling pathways. Targeting these signaling pathways is an attractive strategy to combat I/R injury. In this direction, many investigators have shown that activation of a group of pro-survival protein kinases can offer cardioprotection during I/R injury. These include well-characterized pro-survival protein kinase pathways like Reperfusion Injury Salvage Kinase (RISK), the Survivor Activating Factor Enhancement (SAFE), and the cyclic guanosine 3′,5′-monophosphate/Protein Kinase G (cGMP/PKG) (Perrelli et al., 2011). Among the three, the RISK activation was found to be very effective in combating I/R injury when it is activated before the reperfusion phase. But its prolonged sustained activation leads to the development of hypertrophy, evident from a PI3K transgenic (dnPI3K) mice study, where physiological cardiac hypertrophy was absent when compared with wild type (McMullen et al., 2003). Evidence from the literature emphasizes distinct physiological and pathological events mediated by PI3K activation of its different isoform (Nürnberg and Beer-Hammer, 2019). For instance, p110α, one of the isoforms of PI3K, plays a key role in physiological hypertrophy, whereas p110γ has a crucial role in pathological hypertrophy. In the present study, we administrated fisetin intraperitoneally to the animal before isolating the heart for perfusion, thereby ruling out the possibility of sustained activation of PI3K.

Fisetin was previously reported to modulate PI3K and Akt (one of the downstream targets) in lymphoma and the prostate cancer cell line, respectively. Another downstream target molecule of PI3K is GSK3β, which can also be modulated by fisetin, evident from a rotenone-induced neurotoxicity study. But no study exists with respect to cardiac tissue-specific action of fisetin on the PI3K signaling pathway, where the latter is one of the main cardioprotective signaling pathways in the management of I/R injury. By using an isolated rat heart and LAD model, we confirmed that the underlying mechanism of fisetin-mediated cardioprotection against I/R was associated with the PI3K signaling pathway. In this regard, we used wortmannin, a fungal metabolite that is a potent covalent inhibitor of PI3K (Norman et al., 1996). When we administered wortmannin before the fisetin treatment to the animal before I/R protocol, fisetin failed to improve the decreased I/R associated hemodynamic changes, and increased infarct size, LDH, and CK levels in the blood, and tissue caspase-3 activities both in *ex vivo* and Vivo animal models. At the RNA level, the expression of PI3K mRNA remains low in I/R-challenged fisetin-treated rat heart in the presence of Wortmannin. However, fisetin exhibited an upregulation of PI3K mRNA in the absence of Wortmannin, confirming its interaction in the upstream signaling pathway.

Further, the downstream target of pro-survival signaling at mitochondria was evaluated to reconfirm the result, as mitochondria are one of the key effector targets. Literature showed that cardioprotection mediated by PI3K signaling acts on mitochondria by regulating the membrane permeability transition pore (mPTP) opening. But delay in the mPTP opening is not only mediated by the activation of the PI3K pathway, but also by the inhibition of GSK3β (Juhaszova et al., 2009). Few studies with ischemia-reperfusion rat heart model suggest that the protective effect of PI3K depends on the extend of GSK3β inhibition that prevents the MPTP opening. Accordingly, when we used a GSK3β inhibitor along with fisetin and wortmannin, we found a reversal of negated fisetin-induced cardioprotection, thereby confirming that the underlying mechanism of fisetin is linked to the PI3K/GSK3β signaling pathway, that ultimately protect the mitochondria. This observation is further supported by the improved mitochondrial activity from the I/R challenge in the presence of fisetin.

PI3K signaling pathway activation is reported to regulate the mitochondrial function via inducing peroxisome proliferator-activated receptor *γ* coactivator-1β expression (PGC-1β) (Gao et al., 2011). Similarly, PI3K/Akt signaling can influence the redox homeostasis through its involvement in the activation of both ROS generation and detoxifying processes. But contrary to our expectations, antioxidant enzyme activities were improved and the corresponding lipid peroxidation level was reduced in wortmannin-treated fisetin rat group. This may be due to the free radical scavenging potential of fisetin, as reported by many pieces of literature.

Considering all the above findings and outcome of the experiment, we demonstrated that fisetin could suppress I/R-associated cardiac injury and improve hemodynamics and associated mitochondrial changes through the activation of the PI3K/GSK3β signaling pathway.

## Conclusion

Ischemia-reperfusion injury is one of the critical conditions which requires controlling cell damage as well as protecting organ function. The criticality is exemplified due to its severe clinical manifestations. The current therapeutic interventions are limited, which necessitates the search for new compounds. Plant-based metabolites are well known for their excellent antioxidant activity which has opened avenues for the development of I/R treatment. In this regard, we first identified fisetin, a natural flavonoid, as a possible treatment for I/R. Our results showed that the administration of fisetin can attenuate myocardial ischemia-reperfusion injury by improving the cardiac hemodynamics and reducing cardiac injury in rat heart. This was characterized by enhanced mRNA expression of PI3k and Akt genes and subsequent phosphorylation of PI3k and Akt. By using wortmannin (PI3K inhibitor) before fisetin treatment, the protective effect (elevated cardiac injury and deteriorated hemodynamics) of the molecule was negated. The blockage of PI3K also deteriorated the fisetin-improved mitochondrial function in I/R challenged rats, but not the augmented antioxidant function by fisetin. PI3k-linked fisetin mode of action in rendering cardioprotection was reconfirmed by using GSK3β inhibitor in the presence of wortmannin, where the protective effect of fisetin was reversed. Thus, our results clearly show that PI3K activation is required to mediated fisetin-associated cardioprotection against ischemia-reperfusion injury in rat heart.

## Data Availability

The original contributions presented in the study are included in the article/Supplementary Material, further inquiries can be directed to the corresponding author.

## Glossary

I/R: Ischemia-reperfusion
Akt: Protein kinase B
ATP: Adenosine triphosphate
BSA: bovine serum albumin
CABG: coronary artery bypass graft
cGMP/PKG: cyclic guanosine 3′,5′-monophosphate/Protein Kinase G
CK: creatine kinase
COX: cyclooxygenase
CPCSEA: Committee for the Purpose of Control and Supervision of Experiments on Animals
CT-1: cardiotrophin-1
dnPI3K: dominant negative (dn) PI3K(p110α) mutant
dp/dt: first derivative of the LV pressure
ERK1/2: Extracellular signal-regulated protein kinase
GSH: glutathione
GSK3B: glycogen synthase kinase 3 beta
GSSH: Reduced glutathione
H & E: hematoxylin and eosin
IAEC: Institutional Animal Ethics Committee
IFM: interfibrillar mitochondria
IGF-1: insulin-like growth factor-1
JNK: c-Jun N-terminal kinase
KATP: ATP-sensitive potassium channels
KH: Krebs-Henseleit
LAD: left anterior descending artery
LDH: lactate dehydrogenase
LVDP: left ventricular developed pressure
LVEDP: left ventricular end diastolic pressure
MEK: mitogen-activated protein kinase kinase
MEK1-ERK1/2: extracellular signal-regulated kinase
mPTP: mitochondrial permeability transition pore
NADH: nicotinamide adenine dinucleotide (reduced form)
NADPH: Nicotinamide Adenosine Dinucleotide Phosphate
NQR: rotenone-sensitive NADH-oxidoreductas
OXPHOS: oxidative phosphorylation
p38: mitogen-activated protein kinases
PCR: polymerase chain reaction
PEG, PGC-1B: Proliferator-activated receptor gamma coactivator 1 beta
PI3K: phosphoinositide-3-kinase
PI3K/Akt: phosphoinositide-3-kinase–protein kinase B/Akt
PKA: protein kinase A
PKB/Akt, PKC, PTCA,: percutaneous transluminal coronary angioplasty
QCR: ubiquinol cytochrome c reductase
qPCR: quantitative Polymerase Chain Reaction
RISK: Reperfusion Injury Salvage Kinase
ROS: reactive nitrogen species
RPP: rate-pressure product
SAFE: Survivor Activating Factor Enhancement
SOD: superoxide dismutase
SQR: succinate decylubiquinone DCPIP reductase
SSM: subsarcolemmal mitochondria
TGF-B1: transforming growth factor-B1
TTC: triphenyl tetrazolium chloride
