# Supplementary material for: Fisetin Attenuates Myocardial Ischemia-Reperfusion Injury by Activating the Reperfusion Injury Salvage Kinase (RISK) Signaling Pathway
Source: Front Pharmacol. 2021 Mar 8;12:566470. doi: 10.3389/fphar.2021.566470 (PMC7982788; doi:10.3389/fphar.2021.566470)
Supplement: Supplementary file 1 [file table1.docx]

**Supplementary Table 1.** Cardiac hemodynamic parameters analysis

| **Groups** | **LVDP (x10 mmHg)** | **HR(x10 BPM)** | **LVEDP (mmHg)** |  |
| --- | --- | --- | --- | --- |
| Normal | 11.1 ± 0.9 | 30.0 ± 1.2 | 02 ± 0.40 |  |
| IR | 4.0 ± 1.0^*^ | 24.6 ± 1.9 | 19 ± 0.32^*^ |  |
| Fisetin-Control | 9.8 ± 1.4 | 31.4 ± 1.7**^*^** | 05 ± 0.52**^*^** |  |
| Fisetin-IR | 7.6 ± 0.8 | 28.8 ± 2.4^*^ | 14 ± 0.66 |  |
| Wortmannin-IR | 2.3 ± 0.7^*^ | 11.0 ± 1.8^*^ | 24 ± 0.51^*^ |  |
| Wortmannin-Fisetin-IR | 2.6 ± 0.9^*^ | 14.6 ± 1.2^*^ | 24 ± 0.22^*^ |  |
| Wortmannin-Fisetin-SB216763-IR | 9.6 ± 0.7 | 29.6 ± 2.5**^*^** | 11 ± 0.59 |  |

Data were represented as mean ± SD of 6 individual experiments. L eft ventricular developed pressure (LVDP), Heart rate (HR). Left ventricular end diastolic pressure (LVEDP). * p < 0.05 vs Normal
